# Mevalonate pathway in pancreatic ductal adenocarcinoma: Mechanisms driving metabolic and cellular plasticity

**DOI:** 10.1016/j.cpt.2025.06.004

**Published:** 2025-06-14

**Authors:** Jenna N. Duttenhefner, Katie M. Reindl

**Affiliations:** Department of Biological Sciences, North Dakota State University, Fargo, ND 58102, USA

**Keywords:** Mevalonic acid, Metabolism reprogramming, Pancreatic ductal carcinoma, Molecular targeted therapy, Lipid metabolism, Protein prenylation, Drug therapy, Combination

## Abstract

The mevalonate pathway plays a crucial role in the metabolic reprogramming of pancreatic ductal adenocarcinoma (PDAC), driving lipid biosynthesis, redox homeostasis, and oncogenic signaling, thereby sustaining tumor progression and therapeutic resistance. Its integration with Kirsten rat sarcoma viral oncogene homolog (*KRAS*)-driven signaling networks establishes it as a cornerstone of PDAC biology and a promising therapeutic target. The products of the pathway (sterols and isoprenoids) support key processes such as membrane biogenesis, protein prenylation, and immune evasion, facilitating tumor adaptation to the harsh microenvironment. Despite extensive research, therapeutic resistance and metabolic plasticity present considerable challenges in targeting this pathway. This review synthesizes current knowledge regarding the biochemical regulation of the mevalonate pathway in PDAC, its crosstalk with key oncogenic signaling networks, and emerging therapeutic strategies. In addition, we highlight critical knowledge gaps, including the complex regulatory crosstalk of the pathway with oncogenes, tumor suppressors, and nutrient-sensing pathways, and the mechanisms by which metabolic rewiring modulates tumor–immune interactions and therapy resistance. By integrating insights from pre-clinical and clinical studies, we highlight promising novel combination therapies, including statins, bisphosphonates, and sterol regulatory element-binding protein (SREBP) inhibitors, as well as the potential for precision medicine approaches targeting mevalonate pathway vulnerabilities. Addressing these challenges may provide new avenues for improving therapeutic outcomes in PDAC.

## Highlights

• The mevalonate pathway is a central driver of pancreatic ductal adenocarcinoma (PDAC) biology.
• Kirsten rat sarcoma viral oncogene homolog (*KRAS*) mutations upregulate the mevalonate pathway.
• Mevalonate pathway-derived dolichol supports protein glycosylation, modulating receptor signaling, and tumor adhesion.
• Statins, bisphosphonates, and prenylation inhibitors show promise, especially in combination therapies.

## Graphical abstract

**Figure:**
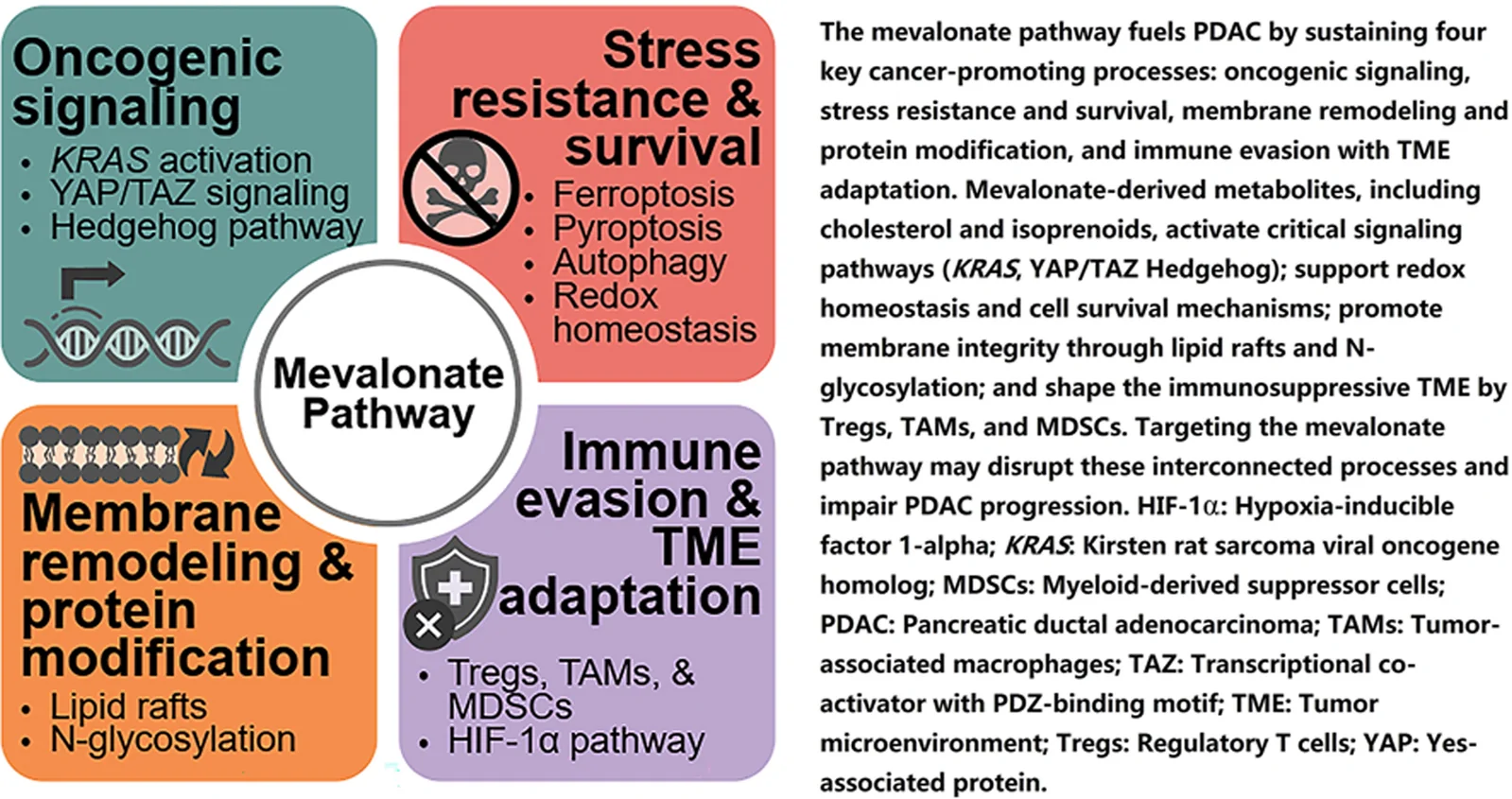

## Introduction

Pancreatic ductal adenocarcinoma (PDAC) is characterized by profound metabolic reprogramming, driving its aggressive behavior and resistance to conventional therapies. Tumor cells exploit these metabolic alterations to thrive in the challenging conditions of the tumor microenvironment (TME), characterized by hypoxia, nutrient deprivation, and oxidative stress. Beyond energy production, these adaptations enhance lipid biosynthesis, redox balance, and anabolic processes essential for tumor growth and survival.^1^ Among these adaptations, lipid metabolism is particularly substantial, as it supports membrane biosynthesis, energy storage, and lipid-mediated oncogenic signaling.^2^ Moreover, central to these processes is the mevalonate pathway, a key metabolic axis that produces sterols and isoprenoids essential for maintaining membrane integrity, redox homeostasis, and post-translational protein modifications.^3^^,^^4^ Mevalonate pathway dysregulation contributes to tumor growth, metastasis, and therapy resistance in various cancers, including PDAC.^3^, ^4^, ^5^ The pathway is tightly linked to Kirsten rat sarcoma viral oncogene homolog (*KRAS*)-driven oncogenic signaling, revealing its fundamental importance in PDAC biology. However, its precise role in coordinating metabolic adaptations and immune evasion remains unclear.^6^, ^7^, ^8^

PDAC exhibits resistance to conventional therapies, which is exacerbated by its dense stroma and unique tumor microenvironment (TME). These characteristics are intricately linked to the tumor’s metabolic adaptations.^9^^,^^10^ PDAC remains one of the deadliest malignancies, accounting for over 90% of pancreatic cancer cases and exhibiting a dismal five-year survival rate of only 13%.^11^ The unique TME of PDAC contributes to this poor prognosis and intrinsic metabolic adaptations, which confer resistance to conventional therapies, enable immune evasion, and accelerate metastasis, often before symptoms arise. Despite the central role of the mevalonate pathway, critical knowledge gaps remain, particularly concerning how this pathway crosstalk with broader metabolic and oncogenic networks in PDAC. Although pre-clinical studies suggest that targeting the mevalonate pathway could enhance therapeutic outcomes, clinical trials have yielded inconsistent results due to metabolic compensation and tumor heterogeneity.^7^^,^^12^, ^13^, ^14^ Addressing these gaps could uncover novel vulnerabilities for therapeutic intervention. This review explores the biochemical mechanisms and regulatory networks governing the mevalonate pathway in PDAC. By exploring its integration with tumor microenvironmental dynamics, metabolic crosstalk, and potential therapeutic targeting, we aim to highlight innovative strategies and future directions for precision-targeted interventions.

## Biochemical overview of the mevalonate pathway

### Key steps in the pathway

**Figure 1:**
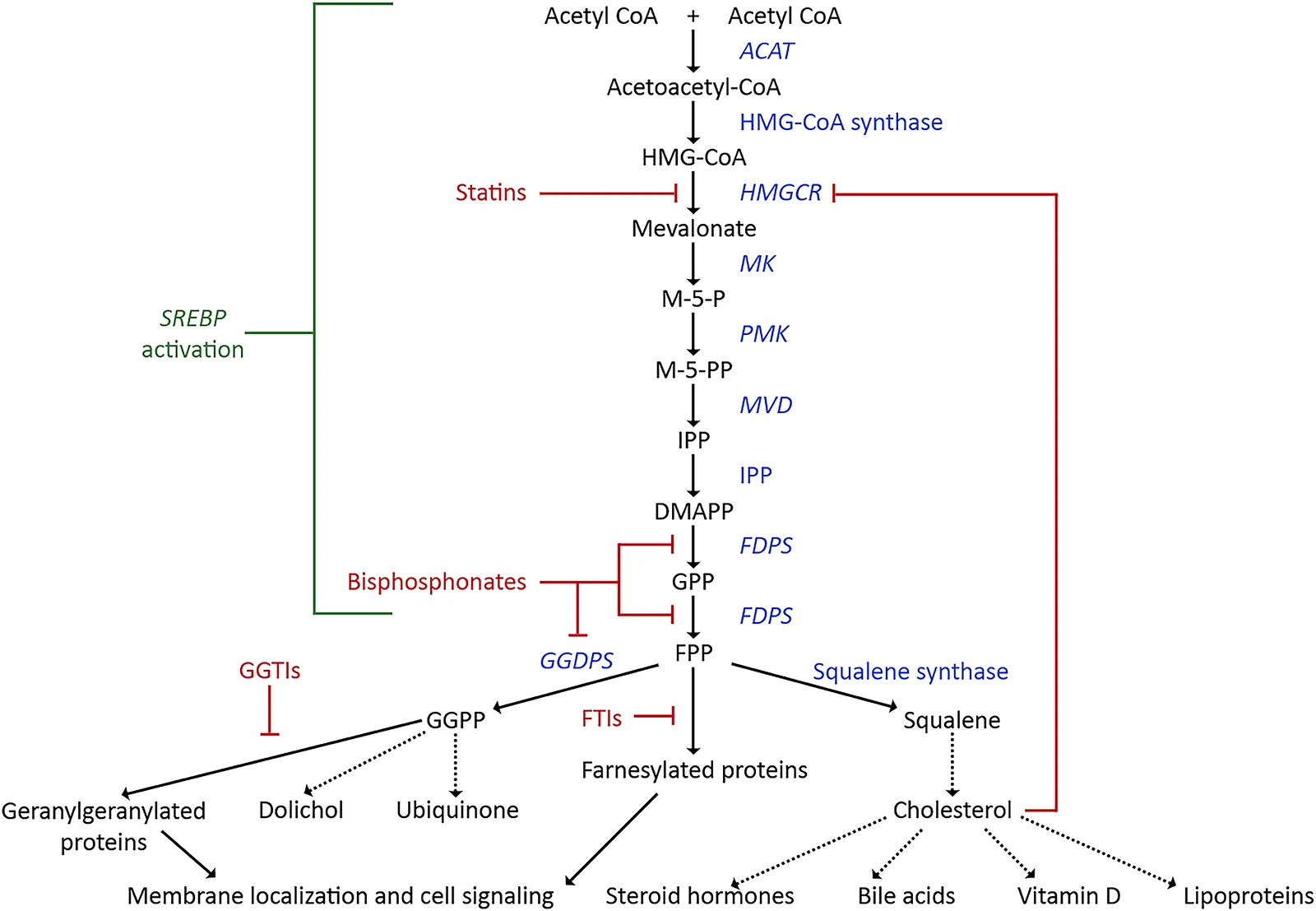
The mevalonate pathway in PDAC: key enzymes, metabolites, and therapeutic targets. The pathway begins with the condensation of two acetyl-CoA molecules to form acetoacetyl-CoA, followed by the synthesis of HMG-CoA synthesis reaction. HMGCR catalyzes the rate-limiting step, converting HMG-CoA to mevalonate. This pathway produces key metabolites, including FPP and GGPP, which are essential for protein prenylation, as well as cholesterol and other downstream derivatives such as steroid hormones, bile acids, and vitamin D. Farnesylated and geranylgeranylated proteins support essential cellular processes, including membrane localization and cellular signaling. Dashed arrows represent multiple steps in the pathway. Several inhibitors targeting this pathway are shown in red. SREBP activation (green) upregulates the expression of mevalonate pathway genes, including HMGCR, MK, and other crucial mevalonate pathway enzymes. ACAT: Acetyl-CoA acetyltransferase; CoA: Coenzyme A; DMAPP: Dimethylallyl pyrophosphate; FDPS: Farnesyl diphosphate synthase; FPP: Farnesyl pyrophosphate; FTIs: Farnesyltransferase inhibitors; GGDPS: Geranylgeranyl diphosphate synthase; GGPP: Geranylgeranyl pyrophosphate; GGTIs: Geranylgeranyltransferase inhibitors; GPP: Geranyl pyrophosphate; HMG-CoA: 3-hydroxy-3-methyl-glutaryl-CoA; HMGCR: HMG-CoA reductase; IPP: Isopentenyl pyrophosphate; M-5-P: Mevalonate-5-phosphate; M-5-PP: Mevalonate-5-pyrophosphate; MK: Mevalonate kinase; MVD: Mevalonate diphosphate decarboxylase; PDAC: Pancreatic ductal adenocarcinoma; PMK: phosphomevalonate kinase; SREBP: Sterol regulatory element-binding protein.

The mevalonate pathway is a tightly regulated metabolic cascade that converts acetyl-coenzyme A (CoA) into sterols and isoprenoids [Figure 1]. The pathway begins with the condensation of two acetyl-CoA molecules to form acetoacetyl-CoA, followed by the conversion to 3-hydroxy-3-methyl-glutaryl-CoA (HMG-CoA) by HMG-CoA synthase. HMG-CoA reductase (HMGCR), the rate-limiting enzyme of the pathway, catalyzes the conversion of HMG-CoA to mevalonate using nicotinamide adenine dinucleotide phosphate (NADPH). This step is tightly regulated and represents a key therapeutic target in cancer.^15^^,^^16^ Downstream of mevalonate, subsequent phosphorylation and decarboxylation reactions yield isopentenyl pyrophosphate (IPP) and dimethylallyl pyrophosphate (DMAPP). Subsequently, a cascade of further condensation reactions by farnesyl diphosphate synthase (FDPS) and geranylgeranyl pyrophosphate synthase (GGDPS) generate the isoprenoid precursors, farnesyl pyrophosphate (FPP) and geranylgeranyl pyrophosphate (GGPP), which are crucial for various cellular functions.^4^^,^^6^^,^^17^^,^^18^

FPP is particularly important for post-translational protein prenylation, a modification that attaches lipid groups to proteins, enabling their membrane association. This modification facilitates the activation of small guanosine triphosphatases (GTPases) such as Ras, which promote many oncogenic processes in cancer cells.^6^^,^^19^ In PDAC, FPP-derived protein farnesylation is critical for *KRAS* protein activation, a hallmark of this malignancy, occurring in approximately 90% of cases.^6^^,^^20^ GGPP, derived from FPP, supports the geranylgeranylation of proteins such as Rho GTPases, which regulate cell proliferation, migration, and survival.^21^ Consequently, *KRAS* mutations upregulate the mevalonate pathway, enhancing *HMGCR* and *FDPS* expression and ensuring a steady FPP and GGPP supply. These modifications underlie the contribution of the pathway to oncogenic signaling, cellular proliferation, and migration.^22^ Disrupting these modifications by inhibiting the mevalonate pathway can impair *KRAS* localization and function, making it a promising therapeutic target. FPP and GGPP also serve as precursors for cholesterol and other important molecules such as ubiquinone and dolichol.

Ubiquinone supports oxidative phosphorylation and adenosine triphosphate (ATP) production and mitigates reactive oxygen species (ROS) as an essential electron carrier in the mitochondrial electron transport chain. In cancer cells, oxidative stress and ATP demand are high owing to increased metabolic activity, making ubiquinone an essential product.^23^ Therefore, the mevalonate pathway contributes to redox balance through ubiquinone synthesis. Dolichol is a long-chain polyisoprenoid lipid involved in glycosylation processes crucial for protein folding, stability, and trafficking. The production of dolichol from FPP is essential for maintaining the proper function of glycoproteins, which fosters cell adhesion and signaling, particularly in tumor progression and metastasis.^24^, ^25^, ^26^ Overall, the mevalonate pathway connects lipid metabolism to key oncogenic signaling networks. These metabolites and precursors link the mevalonate pathway to energy production, glycosylation, and redox homeostasis, establishing this pathway as a central metabolic hub in PDAC and a compelling target for therapeutic intervention.

*De novo* cholesterol synthesis occurs through the mevalonate pathway. Cholesterol is a vital component of cell membranes, contributing to their structural integrity and fluidity. Beyond its role in membrane architecture, cholesterol is also a precursor for synthesizing steroid hormones, bile acids, and vitamins.^5^ Cholesterol is also responsible for modulating signaling pathways, including those involving lipid rafts, which are platforms for receptor clustering and signal transduction.^7^^,^^27^^,^^28^ Cholesterol metabolism is often dysregulated in cancer cells, as increased cholesterol availability supports membrane biosynthesis required for the rapid growth of proliferating tumor cells.^5^^,^^29^

### Regulatory mechanisms

Mevalonate pathway activity is meticulously regulated to balance the cellular demand for sterols, isoprenoids, and other downstream metabolites, ensuring that the pathway responds dynamically to changes in metabolic and environmental conditions, especially in cancer cells. This regulation is orchestrated through transcriptional control by sterol regulatory element binding proteins (SREBPs), feedback inhibition, and integration with nutrient-sensing pathways like (phosphoinositide 3-kinase/protein kinase B (AKT)/mechanistic target of rapamycin) PI3K/AKT/mTOR and adenosine monophosphate (AMP)-activated protein kinase (AMPK). Dysregulation of these regulatory mechanisms amplifies the activity of the pathway in PDAC, facilitating metabolic reprogramming and therapeutic resistance.

The *SREBP* family, particularly *SREBP2*, are the central transcriptional regulators of the mevalonate pathway. Under low cholesterol conditions, *SREBP*s are activated and translocated from the endoplasmic reticulum to the Golgi apparatus, where they are cleaved to release their active nuclear forms. These nuclear *SREBP*s bind sterol regulatory elements (SREs) in the promoters of target genes, such as *HMGCR* and the low-density lipoprotein receptor (*LDLR*), upregulating their expression and increasing cholesterol synthesis and uptake. This process restores cholesterol levels and maintains cellular lipid homeostasis.^30^, ^31^, ^32^ Conversely, as cellular cholesterol levels rise, a feedback mechanism is triggered to reduce *SREBP* activation. Elevated cholesterol levels inhibit *HMGCR* activity by binding to the *SREBP* cleavage-activating protein (SCAP) and insulin-induced gene (INSIG) proteins, which sequester *SREBP*s in the ER, preventing their activation. INSIG facilitates *HMGCR* ubiquitination and degradation, reducing pathway enzyme expression and cholesterol synthesis.^7^^,^^18^^,^^30^ This feedback inhibition ensures that cholesterol production is tightly regulated to prevent excess accumulation, which could be detrimental to the cell.

Cholesterol homeostasis is also controlled through liver X receptor (LXR) activation by oxysterols, metabolites derived from cholesterol oxidation, increasing cholesterol efflux.^7^ Sterol O-acyltransferase 1 (SOAT1) has also been critical in maintaining mevalonate pathway activity by converting free cholesterol into inert cholesterol esters. This conversion prevents free cholesterol accumulation, circumventing the feedback mechanism and sustaining mevalonate pathway activity.^33^ Inhibiting SOAT1 in (tumor protein p53 gene) *TP53*-mutant PDAC cells has been shown to impair cell proliferation and tumor growth, establishing the critical role of this feedback mechanism in PDAC.

Further, the mevalonate pathway does not operate in isolation; it interacts with several nutrient-sensing pathways that help coordinate cellular metabolism in response to nutrient availability. The *PI3K*/*AKT*/*mTOR* pathway is a major regulator of cell survival and proliferation in response to growth factors.^12^^,^^27^^,^^34^^,^^35^ *PI3K*/*AKT* pathway activation by growth factors enhances *SREBP* activation and stabilizes nuclear *SREBP*s by preventing their proteasomal degradation.^7^ This pathway also increases glucose uptake and glycolysis to supply acetyl-CoA and NADPH necessary for mevalonate biosynthesis.^36^ A feedback loop exists wherein mevalonate pathway inhibition reduces phosphatidylinositol-4,5-bisphosphate 3-kinase (*PI3K*) activity, thereby affecting rat sarcoma virus protein (Ras) isoprenylation, emphasizing the integration of growth signals and mevalonate pathway regulation. Downstream of the *PI3K*/*AKT* pathway, the mammalian target of rapamycin (*mTOR*) responds to the availability of amino acids, glucose, and growth factors to promote anabolic processes. *mTOR* signaling promotes *SREBP2* expression, enhancing lipogenesis and supporting the mevalonate pathway through enhanced *HMGCR* expression.^37^ This coordination supports cancer cell proliferation and survival by ensuring an adequate supply of cellular building blocks. Insulin signaling can also modulate the mevalonate pathway by activating the *mTOR* pathway, particularly in cancer cells with aberrant insulin sensitivity.^31^^,^^38^

Beyond promoting biosynthesis, *mTOR* is also crucial in suppressing autophagy and maintaining endoplasmic reticulum homeostasis. In PDAC, where cells experience elevated endoplasmic reticulum stress levels owing to their biosynthetic demands, mevalonate pathway inhibition impairs *mTOR* activity by depleting isoprenoids such as GGPP, which are necessary for activating small GTPases required for autophagosome maturation. This *mTOR* signaling suppression reduces protein synthesis and autophagic flux, leading to misfolded protein accumulation and heightened endoplasmic reticulum stress.^39^ In PDAC, the inability to activate autophagy due to mevalonate pathway disruption may increase tumor cell susceptibility to therapeutic agents.^39^, ^40^, ^41^ This mechanistic link offers a potential therapeutic opportunity to combine mevalonate pathway inhibitors with agents that exacerbate endoplasmic reticulum stress or impair proteostasis to selectively target PDAC cells dependent on endoplasmic reticulum remodeling for survival.

In contrast, *AMPK* functions as an energy sensor to inhibit the mevalonate pathway in response to low energy levels.^4^^,^^42^^,^^43^ *AMPK* phosphorylates *HMGCR*, reducing its activity and suppressing *SREBP*s, impairing lipid synthesis.^4^ Notably, the regulatory role of *AMPK* extends its influence to the *mTOR* pathway, providing a crucial link between energy status and lipid metabolism. Further studies have identified an additional upstream target of the *AMPK* pathway, the tumor suppressor liver kinase B1 (LKB1), whose activity depends on farnesylation mediated by the mevalonate pathway.^44^ This feedback loop showcases the interaction between the mevalonate pathway and energy metabolism, verifying the adaptability of cancer cells to fluctuating metabolic environments.

Recent evidence shows that oncogenic and tumor-suppressive pathways converge at the mevalonate pathway. The tumor suppressor protein, *TP53*, disrupts the mevalonate pathway by blocking *SREBP2* activation.^32^ In contrast, mutant *TP53*, frequently observed in PDAC, interacts with *SREBP2* to upregulate mevalonate pathway genes.^23^^,^^32^^,^^42^ This upregulation in mevalonate pathway products stabilizes mutant *TP53* protein through geranylgeranylation, membrane trafficking, and protection from ubiquitin-mediated proteolysis.^45^ These interactions between *TP53* and the mevalonate pathway indicate that this pathway could serve as a promising therapeutic target for tumors harboring specific *TP53* mutations. Studies have reported that mutated *TP53* interacts with *MYC*, another transcription factor that acts as an oncogene downstream of *KRAS*. *MYC* also influences the mevalonate pathway by binding to *SREBP1* and the promoter regions of mevalonate pathway genes, including *HMGCR*, facilitating mevalonate enzyme expression.^32^^,^^46^, ^47^, ^48^ Further, these regulatory mechanisms form a complex network that allows PDAC cells to fine-tune mevalonate pathway activity in response to metabolic and environmental cues. In PDAC, these connections are amplified by the high prevalence of *KRAS* and *TP53* mutations, which hijack the pathway to support rapid proliferation, immune evasion, and metabolic plasticity. Dysregulation of these control points drives cancer progression, highlighting the therapeutic potential of the pathway.

## Role of the mevalonate pathway in pancreatic ductal adenocarcinoma pathogenesis

The mevalonate pathway is pivotal in PDAC pathogenesis, supplying metabolites for meeting the energetic and biosynthetic demands of proliferating tumor cells. Beyond these roles, mevalonate-derived sterol and isoprenoid metabolites function as critical modulators of downstream signaling pathways essential for tumor progression. These metabolites support membrane biogenesis, protein prenylation, and lipid-mediated signaling, enabling PDAC cells to sustain rapid growth and survival. Notably, key oncogenic pathways, including yes-associated protein (YAP)/transcriptional co-activator with PDZ-binding motif (TAZ) and Hedgehog signaling, considerably depend on mevalonate pathway products to promote tumor proliferation, stemness, and therapy resistance. Cholesterol biosynthesis via the mevalonate pathway contributes to lipid raft formation, stabilizing receptor-mediated signaling and enhancing interactions between oncogenic effectors. These interconnected mechanisms reinforce the role of the pathway in shaping metabolic and signaling networks that drive PDAC progression.

### Protein glycosylation

Protein glycosylation promotes PDAC progression by modulating cell surface interactions, receptor signaling, and tumor cell adhesion.^49^ There are two major types of glycosylation: N-glycosylation and O-glycosylation. N-glycosylation, which is particularly relevant in PDAC, begins in the endoplasmic reticulum with the formation of a lipid-linked oligosaccharide, which is essential for the transfer of glycans to proteins.^50^ This lipid precursor is anchored in the endoplasmic reticulum membrane by dolichol, a molecule synthesized through the mevalonate pathway. Dolichol acts as the carrier for the glycan and is crucial for the proper attachment of the oligosaccharide N-acetylglucosamine (GlcNAc) to the asparagine residue of the protein, forming the core structure needed for N-glycosylation.^50^ The addition and modification of these glycans, which occur as the protein moves through the Golgi apparatus, impact protein stability, folding, and function and regulate numerous cellular pathways crucial for cancer progression.^50^

The mevalonate pathway not only supports lipid metabolism but also influences glycosylation by modulating glycosyltransferase activity. Recent studies have demonstrated the significant role of glycosylation in PDAC chemoresistance, where elevated N-glycosylation contributes to the activation of epithelial–mesenchymal transition (EMT) and survival pathways.^49^ For example, in a recent study, chemoresistant PDAC subtypes showed increased glycosylation and cholesterol metabolism activity. This glycosylation alteration was linked to EMT activation, a process critical for drug resistance and metastasis. The study further highlighted that statins, which inhibit the mevalonate pathway, could significantly reduce protein glycosylation and enhance chemotherapy sensitivity by suppressing EMT signatures in PDAC organoid models.^49^ Disrupting glycosylation through mevalonate pathway inhibition offers a promising therapeutic strategy for overcoming chemoresistance and metastasis in PDAC. Targeting both metabolic and glycosylation pathways may provide a comprehensive approach to improving treatment outcomes for patients with PDAC, particularly those with chemoresistant diseases.

## Yes-associated protein/transcriptional co-activator with PDZ-binding motif signaling activation

**Figure 2:**
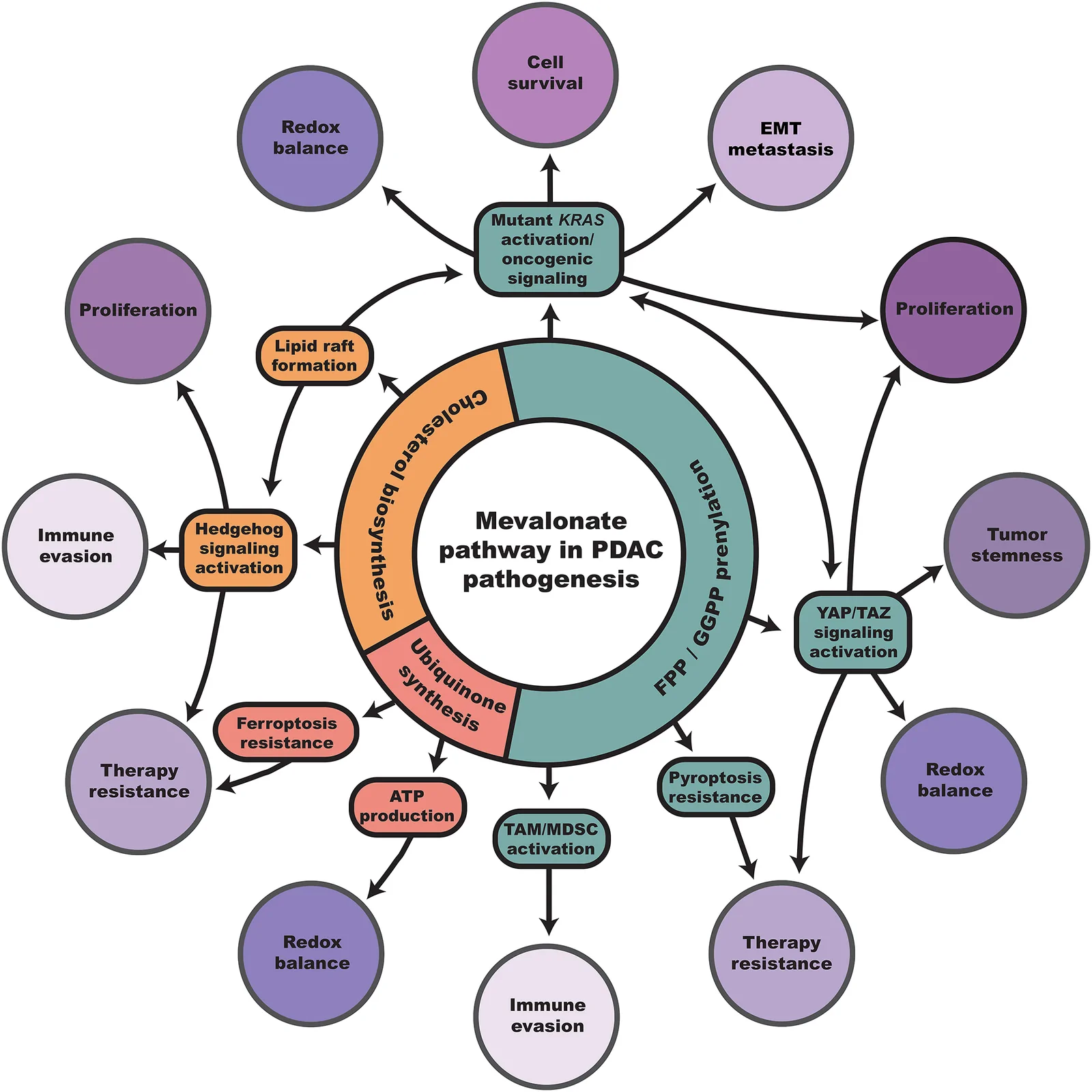
Oncogenic, immunosuppressive, and survival-promoting roles of the mevalonate pathway in PDAC. The mevalonate pathway contributes to PDAC pathogenesis through the biosynthesis of cholesterol, ubiquinone, and isoprenoids (FPP and GGPP). These metabolites support oncogenic processes by promoting mutant *KRAS* signaling, YAP/TAZ activation, lipid raft formation, and immune evasion. In addition to enhancing proliferation, tumor stemness, EMT, and redox balance, the pathway also contributes to ferroptosis and pyroptosis resistance through the regulation of ubiquinone biosynthesis and prenylation of key immune mediators. These processes reinforce therapy resistance and survival in the TME. ATP: Adenosine triphosphate; EMT: Epithelial-mesenchymal transition; FPP: Farnesyl pyrophosphate; GGPP: Geranylgeranyl pyrophosphate; *KRAS*: Kirsten rat sarcoma viral oncogene homolog; MDSC: Myeloid-derived suppressor cell; PDAC: Pancreatic ductal adenocarcinoma; TAM: Tumor-associated macrophage; TAZ: Transcriptional co-activator with PDZ-binding motif; TME: Tumor microenvironment; YAP: Yes-associated protein.

The Hippo pathway effectors YAP and TAZ are critical oncogenes in PDAC progression.^51^, ^52^, ^53^ YAP/TAZ signaling induces transcriptional programs promoting tumor proliferation, stemness, metabolic reprogramming, and therapy resistance, and their dysregulation is common in PDAC [Figure 2].^4^^,^^52^, ^53^, ^54^, ^55^, ^56^ GGPP-mediated geranylgeranylation is critical for actin cytoskeleton remodeling, facilitating the nuclear localization and transcriptional activation of YAP/TAZ by modulating Hippo pathway signaling.^4^^,^^42^^,^^57^ In addition to mevalonate-derived signals, mutant *TP53* also promotes YAP/TAZ activation, further contributing to their nuclear accumulation and downstream oncogenic effects.^42^ Once localized in the nucleus, YAP/TAZ enhances the transcription of genes linked to cell proliferation and therapy resistance, contributing to the aggressive PDAC phenotype.^4^^,^^42^ This signaling axis illustrates how the mevalonate pathway enhances the tumor-initiating properties of PDAC cells via YAP/TAZ activation.

Further, YAP/TAZ activation occurs through both *KRAS*-dependent and *KRAS*-independent mechanisms, highlighting its broad relevance to PDAC pathogenesis. Recent studies suggest that mutations in *KRAS* drive mevalonate pathway activation, enhancing YAP/TAZ signaling and amplifying tumor-initiating properties.^52^^,^^53^ However, recent studies have demonstrated that YAP/TAZ amplification alone is sufficient to bypass *KRAS* dependency and sustain tumor growth.^51^^,^^52^ This finding illustrates the capacity of YAP to function as a *KRAS* substitute in promoting PDAC progression. Despite this, genetic alterations in the Hippo pathway are rare in PDAC, highlighting the need to better understand non-mutational mechanisms regulating YAP activity.

Beyond its role in tumor cells, YAP/TAZ also modulates the PDAC microenvironment, particularly by shaping fibroblast activation and stromal remodeling.^58^ Pancreatic stellate cells, which give rise to most cancer-associated fibroblasts, express high YAP and TAZ levels when activated in PDAC.^58^ YAP promotes the expression and secretion of cytokines and chemokines that facilitate the recruitment and differentiation of myeloid-derived suppressor cells (MDSCs) and tumor-associated macrophages (TAMs), supporting an immunosuppressive TME.^51^ Mechanistically, YAP also functions as a sensor and effector of mechanical and biochemical cues within the tumor niche. The Hippo pathway plays a central role in mechanotransduction, responding to changes in extracellular matrix stiffness, a hallmark of PDAC stroma, and altering cellular behavior accordingly.^54^^,^^59^ Elevated stiffness suppresses Hippo pathway signaling and enhances YAP/TAZ nuclear localization and transcriptional activity, reinforcing pancreatic stellate cell activation and stromal remodeling.^54^ Despite their importance, direct YAP/TAZ pharmacological inhibitors remain elusive. Consequently, therapeutic strategies targeting upstream regulators, such as the mevalonate pathway, are of growing interest. Furthermore, the mechanisms underlying *KRAS*-independent YAP/TAZ activation and their interaction with fibroblasts and the extracellular matrix necessitate future investigation.

### Hedgehog signaling

The Hedgehog signaling pathway, a regulator of embryonic development and tissue homeostasis, is aberrantly activated in PDAC and contributes to tumor growth, desmoplasia, and therapy resistance [Figure 2].^60^^,^^61^ The mevalonate pathway intersects with Hedgehog signaling by providing cholesterol and other sterol intermediates essential for Hedgehog ligand activation and downstream signaling. Cholesterol facilitates post-translational modification of Hedgehog ligands, such as Sonic Hedgehog (*SHH*), enabling their incorporation into cellular membranes and promoting their secretion and activity.^4^^,^^57^^,^^62^ This process is localized to cholesterol-enriched lipid rafts, where Hedgehog receptors cluster and amplify pathway activation.^62^ In addition, aberrant *SHH* expression is strongly associated with oncogenic *KRAS* activity, potentially mediated via nuclear factor kappa-B (*NF-κB*) signaling.^60^ In pancreatic cancer development, ectopic mutant *KRAS*^G12D^ expression directly induced *SHH* transcription, suggesting that *SHH* is a downstream effector of oncogenic *KRAS* signaling. This upregulation is probably mediated through *NF-κB*, a transcription factor constitutively active in PDAC and known to bind to the *SHH* promoter.^60^ This interplay reveals the complex relationship between metabolic reprogramming and oncogenic signaling in PDAC.

Furthermore, the dense stromal microenvironment promoted by Hedgehog signaling impairs drug delivery and fosters immune evasion.^60^^,^^61^ Within the TME, *SHH* acts in a paracrine fashion to activate canonical Hedgehog signaling in cancer-associated fibroblasts.^60^^,^^61^ This tumor–stroma interaction enhances extracellular matrix deposition and creates a fibrotic environment that supports tumor growth and chemoresistance. *SHH*-activated cancer-associated fibroblasts promote tumor proliferation, invasion, metastasis, and gemcitabine resistance.^60^ These findings suggest that dual inhibition of the mevalonate and Hedgehog pathways could disrupt key oncogenic processes, offering a potential therapeutic target. However, this duality of Hedgehog signaling, with both tumor-suppressive and tumor-promoting roles depending on tumor stage and microenvironmental context, complicates its therapeutic targeting and warrants further investigation.

### Cholesterol and lipid raft formation

Lipid rafts, cholesterol-rich microdomains covering 70–80% of the plasma membrane, are crucial in maintaining membrane integrity and supporting signaling pathways [Figure 2].^7^^,^^57^ These dynamic structures compartmentalize and concentrate specific proteins, including oncogenic *KRAS* effectors and survival signaling molecules, thereby facilitating efficient and localized signal transduction. Notably, beyond their relationship with Hedgehog signaling, lipid rafts serve as platforms for the components of the PI3K/AKT pathway, a major regulator of cell proliferation, survival, and metabolism, frequently activated downstream of *KRAS* mutations in PDAC.^27^^,^^63^ Consequently, these disruptions may either promote or inhibit cancer progression, depending on the context.^7^ Cholesterol homeostasis disruption in lipid rafts impairs this pathway, as shown by knockdown models of squalene epoxidase (*SQLE*), a critical enzyme that converts squalene to cholesterol. *SQLE* knockdown reduces raft integrity and suppresses the phosphorylation of key PI3K/AKT players.^27^ Additional regulators such as cellular retinoic acid-binding protein 2 (CRABP-II) influence lipid raft composition by upregulating the (sterol regulatory element binding protein 1c-HMG-CoA reductase-low-density lipoprotein receptor) *SREBP-1c-HMGCR-LDLR* axis, causing chemoresistance to gemcitabine.^63^ Caveolin-1 (CAV-1), a raft-related scaffolding protein, is also upregulated in PDAC and sustains AKT signaling. CAV-1 depletion induces apoptosis and sensitizes cells to chemotherapy.^7^ The role of the mevalonate pathway in orchestrating cholesterol biosynthesis and lipid raft stability highlights its importance in PDAC pathogenesis. Therapeutic strategies targeting lipid rafts or cholesterol biosynthesis may synergize with existing treatments, particularly in combination with immune checkpoint inhibitors or metabolic inhibitors.

### Ferroptosis and pyroptosis regulation

Emerging evidence has linked the mevalonate pathway to the regulation of non-apoptotic cell death pathways, particularly ferroptosis, and pyroptosis, both of which are essential in PDAC biology. These forms of regulated cell death differ from classical apoptosis and contribute to tumor suppression, immune response modulation, and sensitivity to therapy. However, PDAC cells leverage the mevalonate pathway to suppress these processes, thereby promoting their survival and progression. Ferroptosis is an iron-dependent form of cell death driven by lipid peroxidation and impaired redox homeostasis.^64^ Recent studies have revealed that the mevalonate pathway is crucial in ferroptosis regulation through multiple mechanistically distinct avenues.^36^^,^^65^, ^66^, ^67^ First, the synthesis of glutathione peroxidase 4 (GPX4), a key enzyme that suppresses ferroptosis, depends on the availability of a specialized selenocysteine-transfer RNA (tRNA). This tRNA undergoes maturation via isopentenylation, a modification dependent on IPP, a key mevalonate pathway intermediate.^65^^,^^68^ Second, the mevalonate pathway contributes to ubiquinone and squalene synthesis. Ubiquinone and squalene are mevalonate pathway products that act as lipid-soluble antioxidants to sequester lipid peroxidation in a GPX4-independent manner, protecting cells from ferroptotic damage.^65^^,^^69^

Consequently, in PDAC specifically, evidence suggests that cancer cells upregulate the mevalonate pathway to manage oxidative stress and evade ferroptosis and that inhibiting this pathway, either by statins or fatostatin, an *SREBP1* inhibitor, can trigger ferroptotic death.^68^^,^^70^ Statin treatment induces oxidative stress and ferroptosis in multiple PDAC cell lines, although its effects *in vivo* are variable owing to stromal influences that modulate redox signaling.^70^ Fatostatin suppresses proliferation and lipid biosynthesis in PDAC cells while inducing ferroptosis by downregulating GPX4 expression, thereby positioning *SREBP* as a key upstream regulator of ferroptotic sensitivity in these tumors.^68^ Furthermore, targeting downstream enzymes in cholesterol biosynthesis also affects ferroptosis outcomes. For example, inhibition of 7-dehydrocholesterol reductase (DHCR7), a direct precursor to cholesterol, increased ferroptosis resistance.^65^

In contrast, pyroptosis is a pro-inflammatory form of programmed cell death mediated by gasdermin family proteins (GSDMs), particularly gasdermin D (GSDMD), and caspase activation. Although direct pyroptosis regulation by the mevalonate pathway in PDAC remains less defined, indirect mechanisms have been proposed. Recent studies have demonstrated that inhibiting key mevalonate pathway enzymes using statins can trigger pyroptotic cell death and contribute to anti-tumor activity. In pancreatic cancer models, simvastatin partially enhanced 5-fluorouracil (5-FU) efficacy by promoting pyroptosis.^71^ Moreover, the accumulation of mevalonate pathway intermediates such as GGPP may counteract pyroptosis-mediated tumor suppression, highlighting the dual regulatory potential of this pathway. Although emerging evidence connects GSDMD activation to the mevalonate axis, other gasdermins have been implicated in pancreatic tumor progression, suggesting a broader relevance of pyroptosis regulation in PDAC. These findings underscore the complex relationship between the mevalonate pathway, redox homeostasis, and programmed cell death vulnerability in PDAC, where targeting these metabolic dependencies may reveal new therapeutic strategies.

Although considerable progress has been made in understanding the role of the mevalonate pathway in PDAC, several critical knowledge gaps remain. For instance, the differential roles of sterol *vs.* non-sterol metabolites in PDAC progression are not fully elucidated. In addition, the regulatory mechanisms linking *KRAS* mutations to specific mevalonate pathway enzymes, particularly at the post-translational level, require further investigation. The context-dependent duality of Hedgehog signaling and its implications for therapeutic targeting also warrant deeper exploration. Moreover, comparative analysis between PDAC and other cancers could identify unique vulnerabilities within the mevalonate pathway specific to PDAC. In summary, developing therapeutic strategies targeting mevalonate pathway intermediates, combined with YAP/TAZ or Hedgehog signaling inhibitors, represents a promising approach for disrupting the metabolic and signaling networks of PDAC. Addressing these gaps will enhance our understanding of PDAC biology and advance efforts to develop more effective treatments.

## Metabolic crosstalk in the tumor microenvironment

### Immune cell metabolism, the mevalonate pathway, and the tumor microenvironment

The TME in PDAC is an intricate, highly dynamic network comprising cancer cells, stromal cells, immune cells, and extracellular matrix components. This environment is majorly influenced by metabolic alterations that support tumor growth and facilitate immune evasion, establishing a competitive and immunosuppressive landscape.^72^ Within this context, the metabolic crosstalk between cancer cells and TME components is pivotal for PDAC progression. Among the oncogenic drivers, *KRAS* mutations stand out for their role in orchestrating immune suppression and PDAC tumor maintenance.^73^ A key regulator in this interplay is the mevalonate pathway, which modulates tumor and immune cell metabolism through diverse metabolic products. Notably, the pathway influences immune cell activity via the prenylation activity of GGPP, which regulates the activation and migration of T cells and macrophages.^51^^,^^74^^,^^75^ TAMs and MDSCs, enriched in the PDAC-TME, are primarily dependent on these pathways to establish an immunosuppressive microenvironment conducive to tumor growth [Figure 2].^9^^,^^76^^,^^77^

The metabolic demands of PDAC cells often exceed those of surrounding immune cells, depriving them of vital nutrients such as glucose and amino acids.^9^ This nutrient deprivation weakens immune surveillance and enhances immune evasion. In PDAC, cancer cells consume considerable amounts of glucose through aerobic glycolysis, producing lactate and acidifying the TME. This acidic environment not only suppresses the cytotoxic activities of T cells and natural killer (NK) cells but also activates the mevalonate pathway through *SREBP2* activation and nuclear translocation.^78^ This not only promotes lipid synthesis in tumor cells but also reshapes immune cell metabolism, reinforcing the immunosuppressive landscape. In addition to direct metabolic regulation, the mevalonate pathway can also influence the expression of immune checkpoint molecules. For example, hypoxia and metabolic stress within the TME can upregulate programmed death-ligand 1 (*PD-L1*) gene expression in PDAC cells, suppressing anti-tumor immune responses and establishing a feedback loop that perpetuates immune evasion.^55^ The mevalonate pathway substantially influences the TME through cholesterol and isoprenoid synthesis. Cholesterol, essential for forming lipid rafts in cell membranes, facilitates the assembly and activation of signaling molecules crucial for both tumor and immune cells.^79^ By modulating the mevalonate pathway, PDAC cells can reprogram immune cell metabolism to favor an immunosuppressive TME, further promoting tumor growth and immunotherapy resistance.

Regulatory T cells (Tregs) represent another key immunosuppressive population in the PDAC-TME whose function and survival are tightly regulated by the mevalonate pathway.^74^^,^^75^^,^^80^, ^81^, ^82^, ^83^ This pathway supports Treg proliferation and stability through its roles in cholesterol biosynthesis and protein prenylation, modifications essential for signal transduction and Treg survival.^74^^,^^80^ In addition, protein prenylation facilitated by GGPP enables proper membrane localization and signaling of immunomodulatory proteins, further reinforcing the immunosuppressive phenotype of Tregs. As previously mentioned, LKB1 activation depends on prenylation *via* mevalonate-derived isoprenoids. In Tregs, LKB1 promotes functional competency by upregulating mevalonate pathway genes, enhancing proliferation, and suppressing pro-inflammatory cytokines such as interferon-gamma and interleukin-17A, independently of AMPK signaling.^74^

Tregs are present in high numbers in the PDAC-TME, contributing to its immunosuppressive nature and facilitating tumor immune evasion.^83^ These cells exhibit a unique metabolic profile that enables them to persist in the nutrient-depleted conditions typical of PDAC, facilitating their proliferation and function despite the harsh environment. The mevalonate pathway is integral to this adaptation, providing lipid precursors necessary for membrane integrity and energy metabolism, which are critical for Treg survival.^75^ The immunological significance of Tregs in PDAC lies in their ability to suppress effector T cell responses, thereby enabling tumor growth and progression.^82^ Furthermore, the crosstalk between Tregs and other immune cells in the TME, such as MDSCs, further complicates the immune landscape in PDAC. These two populations engage in cooperative interactions reinforcing immunosuppression, underscoring the need for a comprehensive understanding of their metabolic and functional interplay.^83^ The shared reliance of Tregs and MDSCs on the mevalonate pathway highlights a potential metabolic vulnerability that could be leveraged to destabilize the TME and enhance anti-tumor immunity.^82^

### Hypoxia and metabolic reprogramming

Hypoxia, a hallmark of the PDAC-TME, is a key driver of metabolic reprogramming in both cancer cells and immune cells. Under oxygen-deprived conditions, hypoxia-inducible factors (HIFs), particularly HIF-1α, become stabilized and activate transcriptional programs that support glycolysis, fatty acid oxidation, and lipogenesis.^84^ Emerging evidence shows that the mevalonate pathway is directly influenced by hypoxia signaling, as HIF-1α upregulates HMGCR, INSIG-2, and *SREBP* expressions, modulating cholesterol biosynthesis in a tightly regulated feedback loop.^4^^,^^85^^,^^86^ In addition, hypoxia induces a truncated, constitutively active form of squalene monooxygenase, an oxygen-dependent enzyme in cholesterol synthesis, preserving mevalonate pathway flux during hypoxia. This adaptation supports tumor survival by sustaining membrane synthesis and sterol-dependent signaling. Furthermore, the mevalonate pathway contributes to redox homeostasis by producing ubiquinone, a key metabolite for mitigating ROS.^23^ Hypoxia amplifies ROS production, making the mevalonate pathway indispensable for tumor cell survival under these conditions.

These hypoxia-driven changes in the mevalonate pathway also influence immune cell behavior within the TME. For instance, in the TME, TAMs polarize into two distinct phenotypes: pro-inflammatory M1-like macrophages and immunosuppressive M2-like macrophages. Hypoxia drives TAMs toward an M2-like phenotype, which relies on oxidative phosphorylation and fatty acid oxidation for energy production. These pathways are supported by mevalonate-derived intermediates such as FPP and GGPP.^87^, ^88^, ^89^ This metabolic transition facilitates tumor progression by promoting angiogenesis, tissue remodeling, and immune suppression. Further, hypoxia also enhances MDSC accumulation and function, and their immunosuppressive activity is partially dependent on glutamine and oxidative metabolism, both of which are modulated by the metabolic output of neighboring tumor cells.^90^^,^^91^ These pathways enable MDSCs to inhibit T cell activation and suppress immune surveillance mechanisms, further promoting tumor evasion.^92^ The integration of hypoxia-driven signaling and the mevalonate pathway highlights the adaptive capabilities of PDAC cells in the TME. This interplay not only sustains tumor growth but also contributes to therapy resistance, underscoring the need for therapeutic strategies that simultaneously target both pathways.

Notably, pharmacological inhibition of the mevalonate pathway using statins reduces HIF-1α levels, an effect reversible by mevalonate pathway metabolites, highlighting a feedback loop wherein these metabolites stabilize HIF signaling.^4^ This reciprocal regulation emphasizes how immune cells, such as cancer cells, exploit hypoxia-induced mevalonate pathway activity to maintain redox balance, promote immune evasion, and sustain TME remodeling. The intersection of hypoxia and mevalonate pathway activation shapes tumor and immune cell metabolism, promoting immune suppression and therapy resistance. Targeting this crosstalk presents a promising strategy for disrupting metabolic support systems that tumors and their microenvironments depend on for survival.

## Therapeutic targeting of the mevalonate pathway in pancreatic ductal adenocarcinoma

### Mevalonate pathway inhibitors

Statins, well known for their cholesterol-lowering effects via *HMGCR* inhibition, have emerged as promising anticancer agents in PDAC. In addition to inhibiting cholesterol synthesis, statins disrupt critical oncogenic processes by depleting downstream isoprenoid intermediates necessary for protein prenylation, mitochondrial function, and membrane dynamics. These effects collectively impair signaling pathways essential for cancer cell proliferation, survival, and metastasis, including *AKT*, transforming growth factor beta (TGF-β), and *SHH* signaling.^31^^,^^38^^,^^94^ In PDAC models, statins modulate various cancer hallmarks. Specifically, atorvastatin has been found to reduce mutant *KRAS* and *TP53-*driven carcinogenesis by the farnesylated chaperone DnaJ Heat Shock Protein Family (Hsp40) Member A1 (DNAJA1), thereby destabilizing mutant *TP53*.^95^ Other studies highlight statin-mediated induction of cellular stress responses, including TME modulation^96^, ^97^, ^98^, ^99^] and triggering cellular processes such as autophagy,^100^, ^101^, ^102^ ferroptosis,^67^^,^^103^] and pyroptosis,^71^ as well as unfolded protein responses linked to enhanced immunogenicity in *KRAS* mutant tumors.^104^ In a recent investigation, statin treatment activated the c-Jun N-terminal kinase (JNK) pathway, suppressed YAP/TAZ transcriptional activity, and reduced PD-L1 expression, revealing a dual mechanism of growth suppression and modulation in PDAC cells.^55^

Transcriptomic profiling reveals that statins influence several cellular pathways in PDAC cells, extending beyond cholesterol synthesis. Comparative analyses show statin-specific differences in gene expression, lipid metabolism, and stress response pathways, further supporting their multifaceted biological effects.^94^ Statins also impair lipid droplet formation and limit ubiquinone synthesis, thereby enhancing metabolic vulnerability.^105^ In addition, pre-clinical data suggest that statins can counteract the tumor-promoting effects of chronic inflammation and cytokine production. For example, statin use was shown to block interleukin-33 expression, which is implicated in cancer development in pro-inflammatory settings.^106^ However, resistance to statin therapy presents an extensive clinical barrier. Cancer cells may adapt via HMGCR upregulation or activation of alternate cholesterol biosynthesis pathways, diminishing the effectiveness of statins as monotherapies.^16^ This compensatory HMGCR overexpression restores mevalonate flux and prenylation capacity, reducing statin efficacy. Strategies to overcome this include using HMGCR degraders such as SR-12813, which prevent compensatory HMGCR upregulation and augment statin efficacy in PDAC cells.^16^

In clinical settings, retrospective analyses, and randomized trials, reports of the survival benefits of statins remain inconsistent across patient populations, underscoring the challenges posed by the heterogeneity of PDAC and the dense, drug-resistant TME.^93^^,^^94^^,^^107^ A pooled analysis of phase III studies in metastatic PDAC reported modest survival advantages with statin use, though statistical significance was not uniformly achieved.^108^ PDAC subtype stratification is increasingly recognized as essential to tailoring statin-based approaches, given distinct metabolic dependencies that influence treatment response. Large registry-based analyses and retrospective cohorts echo these inconclusive results, with some failing to observe survival benefits among patients with resection or metastasis treated with statins.^109^, ^110^, ^111^ Notably, emerging evidence highlights distinct PDAC subtypes with variable sensitivity to metabolic intervention. A study classified PDAC tumors based on their niche-factor dependencies and demonstrated divergent responses to statin treatment, suggesting that subtype stratification may be critical for optimizing statin-based therapies.^112^ To synthesize recent developments, Table 1 summarizes key *in vitro*, *in vivo*, and clinical study analyses from the past five years evaluating the effects of statins in PDAC. Together, these findings highlight statins as promising yet context-dependent modulators of mevalonate metabolism, whose full therapeutic potential may be realized through rational combinations or patient stratification strategies.

**Table 1:** Overview of the statin-based therapeutic approaches targeting the mevalonate pathway in pancreatic cancer.

Class	Target	Mechanism of action	Drugs	Evidence by study type			Challenges/limitations
				*In vitro*	*In vivo*	Clinical studies^a^	
Statins	HMGCR	Inhibits the rate-limiting enzyme of the mevalonate pathway, reducing cholesterol and isoprenoid synthesis	Simvastatin	22,49,55,71,94,99,104,112,114,133,134	55,70,71,99,104, 133,134	55,108,109,111,115,132	Limited efficacy as monotherapy; potential off-target effects; dose-limiting toxicity in combination
			Atorvastatin	12,49,55,94,95,99,106,114	12,95	49,55,108,109,111,115	
			Pitavastatin	49,94,99,102,106, 114,130	102,106,130	106,109,111,115	
			Fluvastatin	22,55,94,114,131	22,131	109,111,115	
			Rosuvastatin	55,94,106,114		55,108,109,111,115	
			Pravastatin	55,94,114		55,108,109,111,115	
			Lovastatin	49,94		108,109,111,115	
			Cerivastatin	94		109,111	

a Includes cohort studies, case reports, and population studies.Summary of key *in vitro, in vivo*, and clinical studies evaluating the effects of statins in PDAC in the past 5 years. HMG-CoA: 3-hydroxy-3-methyl-glutaryl-CoA; HMGCR: HMG-CoA reductase; PDAC: Pancreatic ductal adenocarcinoma.

Other inhibitors targeting enzymes in the mevalonate pathway, such as FPP and GGPP synthase inhibitors, farnesyltransferase inhibitors (FTIs), geranylgeranyltransferase inhibitors (GGTIs), and SREBP inhibitors, are also being explored [Table 2].^113^ Nitrogen-containing bisphosphonates, such as zoledronic acid, inhibit FPP synthase, reducing both FPP and GGPP levels, thereby disrupting small GTPase prenylation and impairing survival signaling.^35^^,^^114^ In PDAC specifically, bisphosphonates also affect the TME, particularly the fibrotic stroma, by reducing pancreatic stellate cell proliferation and activation.^35^ Although bisphosphonates have demonstrated efficacy in reducing cancer cell proliferation and migration *in vitro* and *in vivo*, analysis of a national cancer database found no significant survival benefit for patients with PDAC treated with bisphosphonates alone.^115^ However, co-administration with statins improved patient survival relative to no treatment.^115^ In an alternative approach, adoptive immunotherapy using zoledronate-activated killer cells has shown promise in patients with incurable pancreatic cancer, offering a potential immunomodulatory angle to bisphosphonate-based strategies.^116^

**Table 2:** Therapeutic strategies targeting the mevalonate pathway downstream of HMG-CoA reductase in pancreatic ductal adenocarcinoma.

Class	Target	Mechanism of action	Drugs	Study type
Bisphosphonates	FDPS/GGDPS	FPP and GGPP synthesis inhibition reduces protein prenylation and oncogenic signaling.	Zoledronic acid	*In vitro*^35^^,^^114^*, in vivo*^35^
			Alendronate	*In vitro*^114^^,^^117^^,^^118^*,* clinical study^115^
			Etidronate	Clinical study^115^
			Ibandronate	Clinical study^115^
			Risedronate	Clinical study^115^
			RAM2061	*In vitro*^120^
			VSW1198	*In vivo*^120^
			RB-07-16	*In vitro*^121^*, in vivo*^121^
			CML-07-119	*In vitro*^121^
FTIs	Farnesyl-transferase	Inhibits protein farnesylation, reducing localization, activation, and downstream signaling.	FTI-277	*In vitro*^22^
			Tipifarnib	*In vitro*^55^^,^^122^^,^^123^
			Lonafarnib	*In vitro*^122^^,^^123^
GGTIs	Geranylgeranyl-transferase	Block geranylgeranylation of proteins, preventing membrane localization, activation, and downstream signaling.	GGTI-298	*In vitro*^22^
			GGTI-2133	*In vitro*^120^
			NHB2005	*In vitro*^120^
*SREBP* inhibitors	SREBP	Inhibiting *SREBP* activation downregulates the transcription of lipogenic and cholesterol synthesis genes.	Fatostatin	*In vitro*,^68^^,^^124^*in vivo*^68^^,^^124^
			Various *SREBP* inhibitors (FDA drug screen)	*In vitro*^125^
			PF-429242	*In vitro*^126^
			25-HC	*In vitro*^126^
			Yarrow SFE	*In vitro*^127^*in vivo*^127^
			Timosaponin A3	*In vitro*^128^*, in vivo*^128^
			Resveratrol	*In vitro*^129^*, in vivo*^129^

Overview of therapeutic approaches targeting key enzymes and regulatory pathways within the mevalonate pathway in PDAC. References indicate supporting pre-clinical and clinical evidence from the past 5 years. 25-HC: 25-hydroxycholesterol; FDA: Food and drug administration; *FDPS*: Farnesyl diphosphate synthase; FPP: Farnesyl pyrophosphate; FTI-277: Farnesyltransferase inhibitor-277; FTIs: Farnesyltransferase inhibitors; GGDPS: Geranylgeranyl diphosphate synthase; GGTI-2133: Geranylgeranyltransferase inhibitor-2133; GGTI-298: Geranylgeranyltransferase inhibitor-298; GGTIs: Geranylgeranyltransferase inhibitors; GGPP: Geranylgeranyl pyrophosphate; HMG-CoA: 3-hydroxy-3-methyl-glutaryl-CoA; HMGCR: HMG-CoA reductase; PDAC: Pancreatic ductal adenocarcinoma; SREBP: Sterol regulatory element-binding protein; Timosaponin A3: Filiferin B or Anemarsaponin A3; Yarrow SFE: Yarrow supercritical fluid extract.

Innovative drug delivery approaches are being developed to overcome the limitations of bisphosphonates. Emerging research in this field has yielded an optimized nanoconjugate of a particular bisphosphonate, alendronate, with natural peptides such as scorpion venom or apamin to enhance cellular uptake and therapeutic targeting. These nanoconjugates significantly increased cell cycle arrest and apoptosis in PDAC cell lines.^117^^,^^118^ In addition, a class of non-bisphosphonate, natural-product-derived FPP synthase inhibitors has been developed to improve cell permeability and tested for cytotoxicity in pancreatic cancer models.^119^ Targeting geranylgeranyl diphosphate synthase (GGDPS) has also emerged as a promising approach. GGDPS inhibition reduced GGPP levels in multiple PDAC cell lines and led to increased apoptosis *in vitro* and reduced tumor growth *in vivo.*^22^^,^^120^ Recent development of C6-substituted pyrazolopyrimidine-based bisphosphonates further expands this approach, with compounds demonstrating potent GGDPS inhibition and anti-tumor efficacy across PDAC, colorectal cancer, and multiple myeloma models.^121^ These novel inhibitors represent a next-generation strategy to more selectively and effectively modulate prenylation in PDAC.

Further studies targeting FPP farnesylation activity have focused on inhibiting farnesyltransferase (FTase), preventing the membrane localization of oncogenic proteins such as *KRAS* and disrupting their downstream signaling pathways. FTIs, such as tipifarnib, were initially developed to target KRAS because of their dependency on farnesylation for membrane association and activity.^122^ Despite their initial promise, the clinical efficacy of FTIs alone has been limited owing to compensatory mechanisms, particularly the ability of KRAS to undergo geranylgeranylation, which maintains KRAS activity even when farnesylation is blocked.^48^ Recent studies have focused on overcoming this redundancy. Dual inhibition of FTase and geranylgeranyltransferase (GGTase) effectively reduced tumor growth in patient-derived xenograft (PDX) models of PDAC.^48^ Combining FTIs with KRAS-G12C or *KRAS*-G12D inhibitors has also shown synergistic effects, potentially breaking down resistance mechanisms in pancreatic cancer.^123^

GGTIs disrupt the geranylgeranylation of proteins such as Rho and Rac, leading to impaired cell cycle progression, migration, and survival.^22^ However, the clinical translation of GGTIs has been hindered by challenges such as poor specificity and systemic toxicity, underscoring the need for next-generation inhibitors or advanced delivery platforms to enhance the therapeutic index and reduce off-target effects. Furthermore, the inherent heterogeneity of PDAC and its complex TME may necessitate combination strategies that pair FTIs and GGTIs with other targeted therapies to achieve substantial clinical outcomes.

SREBP inhibitors represent another promising class of agents that disrupt the mevalonate pathway, enhancing cancer cell susceptibility to therapy. SREBP inhibitors, including fatostatin, have shown pre-clinical efficacy by suppressing tumor growth and reducing pancreatic cancer cell viability *in vitr*o.^68^^,^^124^ Mechanistically, pharmacological inhibition with fatostatin and genetic *SREBP* knockdown suppresses tumor growth through the downregulation of SRY-box transcription factor 9 (SOX9), a key marker in early pancreatic neoplasm development.^124^ In addition, recent high-throughput screening has identified several Food and Drug Administration (FDA)-approved drugs capable of inhibiting *SREBP* activation, offering translational potential for rapid repurposing in PDAC treatment.^125^ Pre-clinical studies have also explored the indirect targeting of SREBPs through their activating protein, SCAP. SCAP inhibition demonstrated significant anti-tumor efficacy, including impaired tumor progression in mouse models, suggesting alternative strategies to modulate SREBP activity *in vivo.*^126^ Furthermore, natural compounds that inhibit SREBP signaling have also shown therapeutic potential in pancreatic cancer. Extracts from common yarrow (*Achillea Millefolium*) reduced pancreatic cancer cell viability by inhibiting *SREBP1* and downregulating lipid biosynthesis and uptake pathways.^127^ Similarly, timosaponin A3 (*Anemarrhena asphodeloides*) and resveratrol have demonstrated anti-tumor effects through *SREBP* inhibition, with resveratrol notably enhancing the efficacy of gemcitabine in pancreatic cancer cells.^128^^,^^129^

Although these findings underscore the potential for targeting the mevalonate pathway, considerable clinical challenges remain. Inhibitors targeting these key mevalonate pathway enzymes in PDAC face limitations, including off-target effects, toxicity, and metabolic heterogeneity. *SREBP*s regulate lipid metabolism in both cancerous and normal tissues, raising concerns about systemic side effects. The inherent heterogeneity of PDAC, including variable metabolic dependencies between patients and even among tumor regions, further complicates treatment. Resistance mechanisms, such as upregulation of alternative metabolic pathways or increased lipid uptake from the TME, may also limit the long-term efficacy of *SREBP*-targeted therapies. Addressing these challenges will require the continued development of highly selective compounds, refined drug delivery technologies, and possibly the use of rational combination strategies tailored to individual tumor profiles.

### Combination therapies

Combination therapies are gaining attention as a strategy to improve the efficacy of mevalonate pathway inhibitors in PDAC [Table 3]. Although statin monotherapy has shown limited clinical benefit, pre-clinical and early clinical studies suggest that pairing statins with chemotherapeutic agents or metabolic inhibitors can yield synergistic anti-tumor effects. For instance, combining pitavastatin with gemcitabine significantly reduced tumor growth both *in vitro* and *in vivo*, compared with either agent alone.^102^^,^^130^ Similarly, co-treatment with 5-FU and statins, such as simvastatin or fluvastatin, has demonstrated enhanced efficacy through modulation of cell death pathways, including pyroptosis.^71^ Fluvastatin has also been shown to sensitize PDAC cells to radiation therapy and inhibit tumor-associated fibrosis, thereby overcoming a major hurdle in radioresistance and stromal protection.^131^ Ongoing clinical efforts are now testing statin-based combination therapies in patients. In this phase II study, valproic acid is combined with simvastatin and standard chemotherapies, highlighting the translational potential of epigenetic and metabolic co-targeting in advanced PDAC.^132^ Although these combinations can address the key limitation of statins in PDAC monotherapies, the risk of increased toxicity or resistance remains, necessitating further investigation.

**Table 3:** Combination therapies targeting the mevalonate pathway in pancreatic ductal adenocarcinoma.

Class	Drugs	Findings
Chemo/radiation therapy + statins	Various chemotherapies + statins^49^	Synergistic effect *in vitro* and *in vivo*
	Chemotherapy + atorvastatin^49^	Phase II clinical study - significant tumor reduction
	Oxaliplatin + simvastatin^55^^,^^104^	Synergistic effect *in vitro*^55^^,^^104^ and *in vivo*^104^
	5-fluorouracil + simvastatin^71^	Synergistic effect *in vitro* and *in vivo*
	Gemcitabine + pitavastatin^130^	Synergistic effect *in vitro* and *in vivo*
	Gemcitabine/nab-paclitaxel + simvastatin/valproic acid^132^	Synergistic effect *in vitro* and *in vivo* Ongoing phase II clinical study
	Gemcitabine/paclitaxel + simvastatin/metformin (AMPK inhibitor)/digoxin (PI3K/AKT inhibitor)^134^	Synergistic effect *in vitro* and *in vivo*
	Radiation + fluvastatin^131^	Synergistic effect *in vitro* and *in vivo*
*SREBP* inhibition + statins	TMDP + fluvastatin/simvastatin^22^	Synergistic effect *in vitro*
	PF-429242 + fluvastatin^22^	Synergistic effect *in vitro*
Signaling modulation + statins	SP600125 (JNK inhibitor) + simvastatin^55^	Antagonistic effect *in vitro*
	SB 203580 (p38 inhibitor) + simvastatin^55^	No effect *in vitro*
	BP0273 (PD-1 inhibitor) + simvastatin^55^	Synergistic effect *in vitro* and *in vivo*
	AZD6244 (MEK inhibitor) + simvastatin^99^	Synergistic effect *in vitro* and *in vivo*
	Metformin + pitavastin^102^	Synergistic effect *in vitro*
	Metformin + simvastatin^133^	Synergistic effect *in vitro* and no effect *in vivo*
Bisphosphonates + statins	Various bisphosphonates + statins^115^	No effect clinical study
Ferroptosis induction + statins	Dibenzyl diselenide (DBDS) + simvastatin^70^	Synergistic effect *in vitro* and *in vivo*
Acetyl-CoA synthesis inhibition + statins	JQ1 (ACLY inhibitor) + atorvastatin^12^	Synergistic effect *in vitro* and *in vivo*
Chemo/radiation therapy + bisphosphonates	Radiation + zoledronic acid^35^	Synergistic effect *in vitro* and *in vivo* Ongoing phase I/II clinical study
Chemotherapy + FTI	FOLFIRINOX + tipifarninb^49^	No effect *in vitro*
*KRAS* inhibitors + FTIs	MRTX1133 (KRAS-G12D) + tipifarnib/lonafarnib^122^	Synergistic effect *in vitro*
	Sotorasib/adagrasib (KRAS-G12C) + tipifarnib/lonafarnib^123^	Synergistic effect *in vitro* and sensitization effect *in vivo*
*SREBP* inhibition + FTI/GGTIs	TMDP + FTI-277^22^	Synergistic effect *in vitro*
	TMDP + GGTI-298^22^	Synergistic effect *in vitro*
Dual FTI/GGTI	FGTI-2734^48^	Synergistic effect *in vitro* and *in vivo*
Chemotherapy + *SREBP* inhibition	Gemcitabine + resveratrol^129^	Sensitization effect *in vitro* and *in vivo*

Summary of pre-clinical studies exploring combination therapies involving statins, bisphosphonates, FTIs, GGTIs, and *SREBP* inhibitors in the past 5 years. 5-FU: 5-fluorouracil; ACLY: ATP-citrate lyase; AKT: Protein kinase B; AMPK: AMP-activated protein kinase; AZD6244: Selumetinib; CoA: Coenzyme A; DBDS: Dibenzyl diselenide; FGTI: Farnesyl/geranylgeranyl transferase inhibitor; FOLFIRINOX: Fluorouracil + Leucovorin + Irinotecan + Oxaliplatin; FTI: Farnesyltransferase inhibitor; GGTIs: Geranylgeranyltransferase inhibitors; JNK: c-Jun N-terminal kinase; KRAS: Kirsten rat sarcoma viral oncogene homolog; MEK: Mitogen-activated protein kinase kinase; PD-1: Programmed cell death protein 1; PDAC: Pancreatic ductal adenocarcinoma; *PI3K*: Phosphoinositide 3-kinase; SB 203580: Adezmapimod; *SREBP*: Sterol regulatory element-binding protein; TMDP: Threonyl muramyl dipeptide.

Beyond cytotoxic agents, combinations with targeted therapies also hold promise. Statins have been paired with mitogen-activated protein kinase kinase (MEK) inhibitors (including selumetinib [AZD6244]), demonstrating synergy in KRAS-driven tumors by disrupting oncogenic signaling and enhancing oxidative stress.^99^ Combination studies with statins and immune checkpoint inhibitors, such as programmed cell death protein 1 (PD-1) inhibitors, suggest that statins may modulate the TME to enhance immune responses,^55^ representing a shift toward targeting cancer cell biology and tumor interactions with the immune system, which is critical in overcoming the immune evasion seen in PDAC.

The combination of statins with autophagy inhibitors represents another innovative direction. Cancer cells often rely on autophagy to survive nutrient stress induced by mevalonate pathway disruption. The combined inhibition of both pathways has demonstrated enhanced cytotoxicity in PDAC models, particularly when agents such as pitavastatin are used alongside autophagy modulators such as metformin.^102^ Studies indicate that low-dose metformin and simvastatin co-treatment also suppress PDAC development *in vivo*, supporting the use of metabolic repurposing in combination strategies.^133^ Other repurposing studies have investigated triple combinations, such as metformin, simvastatin, and digoxin, targeting PDAC through multiple mechanisms, including AMPK activation, HMGCR inhibition, and PI3K/AKT inhibition.^134^ Strategies exploiting ferroptosis have also gained momentum. Studies exploring dibenzyl diselenide (DBDS) and simvastatin demonstrate increased tumor cell vulnerability through enhanced ROS production and iron metabolism disruption.^70^ Similarly, inhibition of ATP-citrate lyase (ACLY), which generates acetyl-CoA upstream of the mevalonate pathway, has been shown to sensitize PDAC cells in combination with atorvastatin and may offer another metabolic advantage.^12^

Bisphosphonates, commonly used for managing bone metastases, also exhibit synergistic effects when combined with statins or mevalonate pathway inhibitors.^115^^,^^121^ Combined with FTIs, KRAS inhibitors are emerging as compelling opportunities to target *KRAS*-driven signaling more effectively in PDAC.^122^^,^^123^ Dual prenylation inhibition strategies, such as combining FTIs with GGTIs, have also demonstrated pre-clinical promise. Agents such as farnesyl/geranylgeranyl transferase inhibitor (FGTI-2734) or FTI-277 with GGTI-298 aim to block dual prenylation pathways, further impairing membrane localization and function of oncogenic proteins, such as KRAS in PDAC, providing an effective multi-targeted strategy.^22^^,^^48^ Another emerging tactic involves disrupting lipid biosynthesis through *SREBP* inhibition. Agents such as resveratrol have been shown to enhance gemcitabine sensitivity and reverse cancer stem-like phenotypes via *SREBP1* suppression.^129^

The challenge of targeting the mevalonate pathway in PDAC remains considerable because of the complexity and redundancy of the metabolic processes involved. Tumor cells are adept at compensating for disruptions in key metabolic pathways, leading to a limited therapeutic impact when using single agents. The evolving field of combination therapies offers a promising strategy to overcome this issue. However, further research is needed to optimize treatment regimens and minimize potential toxicities. Synthetic lethality, which targets specific genetic vulnerabilities unique to cancer cells, disrupts two genes whose combined inhibition leads to cell death while sparing normal cells. By identifying metabolic dependencies in PDAC and other cancers, synthetic lethality offers a highly specific mechanism for targeting the mevalonate pathway. CRISPR (clustered regularly interspaced short palindromic repeats) technology has become instrumental in uncovering these dependencies, enabling the identification of less-studied enzymes within the pathway that could serve as viable therapeutic targets.^22^^,^^33^^,^^135^ Moreover, balancing therapeutic efficacy with tolerability will require advanced strategy systems, such as drug delivery systems that specifically target tumors, minimizing off-target effects. Innovative drug delivery systems, such as nanoparticle-based therapies, are also being developed to improve the specificity and efficacy of mevalonate pathway inhibitors. In addition, encapsulating statins or other inhibitors within nanoparticles allows for targeted delivery to tumor cells, reducing systemic exposure and minimizing off-target effects. These advancements hold substantial promise for enhancing the therapeutic index of pathway inhibitors while mitigating adverse effects.

### Gaps in knowledge

Despite growing interest in combination strategies targeting the mevalonate pathway in PDAC, several key limitations continue to hinder the clinical translation of these strategies. Many promising therapeutic combinations remain confined to pre-clinical models, with limited advancement into clinical trials. This gap reflects the inability of *in vitro* and murine systems to fully capture the complexity of the PDAC-TME and the extensive metabolic heterogeneity observed in patients. Moreover, although combination therapies may enhance efficacy, they also increase the potential for systemic toxicity and off-target effects. Addressing these challenges requires careful dose optimization and the development of tumor-targeted delivery systems, such as nanoparticles, to maximize benefit while minimizing harm. Another major obstacle is the inherent metabolic plasticity of PDAC cells. These tumors can rapidly rewire their metabolic networks to bypass the therapeutic inhibition of the mevalonate pathway, thereby diminishing treatment durability and fostering resistance. This issue is compounded by the current lack of predictive biomarkers to identify patients who will most likely respond to mevalonate-targeted therapies or drug combinations. Biomarker discovery remains a high priority in guiding patient selection and therapeutic monitoring.

Further, the field has primarily focused on well-characterized enzymes such as HMGCR and GGDPS, while other nodes in the pathway remain unexplored. Uncovering novel vulnerabilities may require high-throughput functional screens, such as CRISPR-Cas9-based approaches, to systematically interrogate the pathway. Furthermore, emerging evidence suggests that the mevalonate pathway intersects with other stress-adaptive mechanisms, including autophagy and ferroptosis, though the nature of these interactions in PDAC remains unclear. As these processes contribute to cell survival and therapy resistance, understanding their crosstalk with mevalonate signaling is essential for designing effective and durable interventions. These limitations underscore the importance of integrative, system-level approaches that leverage multi-omics data, functional genomics, and patient-derived models. Advancing our understanding of how mevalonate pathway modulation interfaces with other PDAC hallmarks, such as redox regulation, immune evasion, and EMT transition, will be critical for translating pre-clinical findings into meaningful clinical progress and achieving personalized treatment for patients with chemoresistant pancreatic cancer.

## Conclusions

The mevalonate pathway is a central driver of PDAC biology, facilitating major processes such as sterol biosynthesis, isoprenoid production, and oncogenic signaling modulation. These functions support membrane integrity, post-translational protein modifications, and tumor cell survival in the nutrient- and oxygen-deprived TME. Although its critical role in PDAC progression and resistance to therapies has been well-established, considerable gaps remain in fully understanding the mechanistic intricacies of this pathway. Current knowledge indicates how mevalonate pathway dysregulation intersects with other metabolic networks and oncogenic drivers, such as KRAS mutations, to facilitate tumor growth, metastasis, and therapeutic resistance. However, the precise molecular mechanisms governing these interactions and how they contribute to metabolic reprogramming in PDAC remain elusive. Consequently, unraveling these complexities is essential for refining therapeutic strategies and identifying novel vulnerabilities in this pathway.

Advancing research also requires developing robust diagnostic and prognostic tools that reflect the activity of the pathway in PDAC. Biomarkers enabling patient stratification and personalized treatment approaches will be pivotal in optimizing the efficacy of pathway-targeted therapies. In addition, improved pre-clinical models, such as patient-derived organoids and genetically engineered mouse models, are needed to accurately recapitulate the TME and metabolic heterogeneity of PDAC, facilitating the translation of findings from bench to bedside. Emerging therapeutic strategies, including dual inhibition of pathway enzymes, synthetic lethality approaches, and nanoparticle-based delivery systems, show promise for targeting the mevalonate pathway. These approaches aim to enhance pathway suppression while minimizing systemic toxicity, representing a crucial step toward overcoming the formidable resistance of PDAC to treatment. Moreover, integration with complementary therapies, such as autophagy inhibitors or immunotherapies, may further exploit metabolic dependencies within PDAC cells, broadening the therapeutic landscape.

In summary, the mevalonate pathway represents a major component of the metabolic reprogramming of PDAC and a compelling target for therapeutic innovation. Addressing the remaining mechanistic gaps, developing predictive biomarkers, and employing advanced pre-clinical models will facilitate the clinical applicability of research findings. By integrating these strategies, we can harness the full potential of the mevalonate pathway to develop effective therapies against PDAC, ultimately improving outcomes for patients battling this devastating disease.

## Authors contribution

Duttenhefner, Jenna N.: Conceptualization, investigation, visualization, writing - original draft, writing - review & editing; Reindl, Katie M.: Conceptualization, supervision, writing - review & editing. All authors read and approved of the final manuscript.

## Ethics statement

None.

## Data availability statement

The datasets used in the current study are available from the corresponding author upon reasonable request.

## Declaration of generative AI and AI-assisted technologies in the writing process

The authors declare that generative artificial intelligence (AI) and AI-assisted technologies were not used during the preparation of this manuscript. The authors take full responsibility for the content of the publication.

## Funding

None.

## Conflict of interest

The authors declare that they have no known competing financial interests or personal relationships that could have appeared to influence the work reported in this paper.
